# Maternal depression and anxiety disorders (MDAD) and child development: A Manitoba population-based study

**DOI:** 10.1371/journal.pone.0177065

**Published:** 2017-05-24

**Authors:** Brenda Comaskey, Noralou P. Roos, Marni Brownell, Murray W. Enns, Dan Chateau, Chelsea A. Ruth, Okechukwu Ekuma

**Affiliations:** 1 Department of Community Health Sciences, University of Manitoba, Winnipeg, Manitoba, Canada; 2 Manitoba Centre for Health Policy, Winnipeg, Manitoba, Canada; 3 Department of Psychiatry, University of Manitoba, Winnipeg, Manitoba, Canada; 4 Department of Pediatrics, Section of Neonatology, University of Manitoba, Winnipeg, Manitoba, Canada; National Institute of Child Health and Human Development, UNITED STATES

## Abstract

**Objective:**

To examine the association between maternal depression and anxiety disorders (MDAD) and child development assessed during the kindergarten year.

**Methods:**

Administrative data from several health and social databases in Manitoba, Canada, were used to study 18,331 mother-child pairs. MDAD over the period from one year prior to the child's birth to the kindergarten year was defined using physician diagnoses and filled prescriptions. Child development was assessed during the kindergarten year using the Early Development Instrument (EDI) which measures vulnerability across five domains of development. Structural equation modeling was used to examine associations between timing, recurrence and severity of MDAD and child outcomes. Health at Birth (preterm, low birth weight, neonatal intensive care stay and long birth hospitalization), Family Context (teen mother, lone parent, socio-economic status (SES)), child age and child sex were covariates.

**Results:**

MDAD had a modest negative association with child EDI scores across all models tested, particularly for social, emotional and physical development. Prenatal MDAD had a stronger negative association with outcomes than other time periods; however, recurrent MDAD had a stronger negative association with outcomes than any specific time period or MDAD severity. The influence of MDAD was mediated by Family Context, which had a strong, negative association with outcomes, particularly language and cognitive development.

**Conclusion:**

The number of time periods a child was exposed to MDAD in early childhood was more negatively associated with five areas of child development than timing or severity. Prenatal exposure may be more sensitive to MDAD than other time periods. The familial context (teen mother, lone parenthood and low SES) had a stronger influence on child outcomes than MDAD. Findings can be used to inform interventions which address maternal mental health from the prenatal period onward, and to support disadvantaged families to encourage healthy birth outcomes, early childhood development and school readiness.

## Introduction

Early life experiences can “get under the skin” and influence the developing brain, stress response systems and later health outcomes, particularly during sensitive periods[1,2] and early child development has been identified as an important determinant of health.[3] Exposure to maternal depression and anxiety disorders (MDAD) has been shown to have a negative influence on child development in infancy,[4] early childhood,[5, 6] and at school entry,[7, 8] and is associated with poor birth outcomes,[4] elevated stress response,[4, 9, 10] negative temperament,[11] social, emotional and behavioural problems, [12, 13] impaired cognitive performance[14] and compromised physical health.[5] What is less is clear are the mechanisms through which MDAD influences child development.

Possible pathways include: *biological*, such as developmental or fetal programming, [4,15,16] epigenetic processes; [2, 9, 10] and birth outcomes; [17–20] and *family/social context*, including maternal sensitivity;[21–23] and socioeconomic factors such as low income, lone parenthood and teen motherhood.[24] These influences may be mutual or bi-directional [25] and have been found to mediate the relationship between MDAD and child outcomes.[4, 23–26] Further, mechanisms may differ depending on the aspect of MDAD the child is exposed to. Sensitive periods of development for children have been identified based on the *timing* of exposure,[27–29] and poor outcomes have been found where MDAD has been *chronic or recurring*[30, 31] or more *severe*.[31]

School readiness has been identified as a multi-dimensional concept that includes language and cognitive development, social competence, emotional maturity, physical health and communication skills and general knowledge.[32] School readiness is important for later school success and for health, social and economic outcomes over the life course. Children who are not ready for learning at school entry are more likely to repeat grades and receive remedial and special education services.[33] Children with good verbal, social and attention skills are more likely to perform better in school than children without these skills [34, 35] and gaps present at kindergarten[18, 36] tend to widen over time.[37]

Studies that have examined the relationship between MDAD and child outcomes typically rely on maternal symptom reporting, measurement at specific points in time and modest sample size. Few studies have examined the timing, recurrence and severity of MDAD and how these features alone or in combination influence child development. In addition, studies are usually limited to one or two domains of child health, typically emotional and behavioural development. The present study examined the relationship between MDAD and child development, controlling for several health and social factors. Unique features of the present study are a very large population-based sample, the examination of key features of MDAD including timing, recurrence and severity and their differential influence on child development, and the measurement of five different domains of child development at kindergarten.,

## Methods

This longitudinal, population-based study used a retrospective cohort design to examine the relationship between MDAD and child school readiness.

### Data sources

Data are from the Population Health Research Data Repository (“the Repository”) housed at the Manitoba Centre for Health Policy (MCHP) which contains information on all residents of the Canadian province of Manitoba (population 1.3 million). The Repository includes numerous health and social databases of information collected through government departments and linkable using anonymized individual identifiers. For this study, health databases (see Table 1) include physician visits for MDAD, filled prescriptions for anxiety or depression and infant health outcomes from the birth hospitalization record. Age of mother at first birth and marital status were obtained from the Manitoba health insurance registry. Child development scores were obtained from the Early Development Instrument, a tool which measures five domains of school readiness part way through the kindergarten year. Data from the national census included average household income and neighbourhood rates of unemployment, high school completion and lone parenting.

**Table 1:** Data sources used in the study.

Data File	Description
*Population Registry*	Information on all Manitoba residents eligible for receipt of health services in the province
*Medical Services*	Information on ambulatory physician visits, including 3-digit International Classification of Diseases (ICD) codes
*Hospital Discharge Abstracts*	Information on birth hospitalizations, including up to 25 ICD codes of discharge diagnoses
*Prescription Drug Claims*	Information on all prescription medications dispensed outside hospital
*Early Development Instrument (EDI)*	Population-based information on five domains of child development
*Canada Census*	Area-level (neighbourhoods of 400–700 individuals) social and demographic information

### Study population

Children born between January 1, 2000 and December 31, 2001 with a valid EDI score in their first year of kindergarten (2006 or 2007) were linked to their birth mother using an anonymous identifier from the birth record–the personal health information number (PHIN). Health insurance coverage is extended to individuals residing in Manitoba and only mother-child pairs with continuous health insurance coverage for the duration of the study period (from one year prior to birth up to the EDI assessment) were retained. A total of 23,236 mother-child dyads were potentially available from the 2006 and 2007 EDI cohorts. Exclusions for this study include:

- Mother or child missing personal health information number (PHIN) (n = 637);
- Mother or child without continuous Manitoba health coverage (e.g., moved out of province for one or more periods of time) for the study period (n = 3909);
- Children who were younger than age 5 or older than age 6 at the time of the EDI (n = 22);
- Duplicate EDI records (n = 70);
- Children not born in hospital (n = 267).

The final sample was 18,331 mother-child pairs, some of which included multiple children (n = 1547) from the same mother: singleton births (n = 1051); twins (n = 228 pairs) and triplets (n = 4 sets). As each mother-child pair was an independent observation, multiples were not excluded from the study.

### Time periods

Mothers’ contacts with the health care system for depression and anxiety disorders (physician visits and filled prescriptions) were tracked continuously over a six-year period from one year prior to the child’s birth to approximately six months into the kindergarten year (February/March 2006 and 2007). Four discrete time periods were examined: Prenatal year, postnatal year, toddler period, and year before the EDI (See Table 2 for definitions). Models were run on the full sample for each time period and for the overall study period to examine the relationship of timing, recurrence and severity of MDAD to each child’s development.

**Table 2:** Time periods used in the study.

Time Period	Description
Prenatal year	365 days prior to the birth of the child, excluding the date of birth.
Postnatal year	Date of birth plus 364 days, ending the day before the child's first birthday.
Toddler period	Day of the child's first birthday up to one day before the beginning of the year before the EDI (children are different ages at the time of the EDI so the Toddler period is variable in length (mean = 3.7 years or 45 months).
Year before EDI	365 days before the EDI, excluding the assessment date.
Overall study period	All four periods combined. For some analyses (MDAD recurrence and severity), the overall study period (from one year before the birth until the EDI) is the reference period with no further breakdown by time period.

### Variables

#### Definitions

The outcome measures for this study were child scores on five domains of child development as measured by the Early Development Instrument (EDI). Developed by Janus and Offord,[32] the EDI is a validated, 104-item assessment administered by teachers mid-way through the kindergarten year and measures five areas of child development: physical health and well-being; social competence; emotional maturity; language and cognitive development; and communication skills and general knowledge.

Development of the EDI included extensive psychometric testing. Factor analysis of data from over 16,000 kindergarten children[38] grouped 14 factors into the five domains, based on a conceptual framework. The 14 factors accounted for 63.1% of the variance, with social competence accounting for nearly one-third (32.9%). Cronbach’s alpha for the five domains ranged from 0.84 to 0.96, indicating satisfactory internal consistency. Multi-level confirmatory factor analysis revealed that the factor structure within classrooms is similar to the structure between classrooms.[32] Factor analysis across several jurisdictions—Canada, Australia, Jamaica and the US—has found high consistency in factor loading.[39] Assessment of differential item functioning (DIF) with 43,900 children found that substantial DIF effect size on several items was due to true group differences rather than teacher bias.[40] Further, inter-rater reliability correlations range from moderate (0.53) to high (0.80)[32] and the EDI has been found to be valid and unbiased across diverse populations, including gender, language and Aboriginal groups. [38, 40–42] Testing of concurrent validity found that the EDI to be as predictive of school achievement as measures that are more time- and resource-intensive[43] and research in British Columbia has found EDI results to be predictive of performance in grade 4.[44] The EDI outcome values are domain scores out of 10.

Given the richness of the data available, we were able to develop key independent variables using three latent constructs, “unobserved” variables that represent concepts that cannot be measured directly or that any single measure cannot measure well:[45] a) MDAD; b) Health at Birth (based on the work of Fransoo et al.[19]); and c) Family Context. Detailed definitions of the latent constructs are in Table 3.

**Table 3:** Latent constructs used in the statistical modeling.

Latent Construct	Indicator	Definition
***Maternal Depression and Anxiety Disorders (MDAD)***	Antidepressants^†^	Number of mothers' antidepressant prescriptions (ATC code N06A).
	> 1.0 defined daily dose (ddd)	Binary dose-intensity variable indicating if mother had > 1.0 ddd of antidepressants. Defined daily dose (DDD) is an approximation of drug consumption and is an assumed average dose per day for particular solid-form drugs when used for their primary indication.[46] See also: http://mchp-appserv.cpe.umanitoba.ca/viewDefinition.php?definitionID=102536.
	Sedatives/Hypnotics^†^	Number of mothers' sedative/hypnotic prescriptions (ATC codes N05B and N05C).
	Physician visits^†^	Number of mothers' depression or anxiety physician visits (ICD-9 codes 296, 300, 309, 311).
***Health at Birth***	Low birth weight	Binary variable indicating infant’s birth weight was <2500 grams.
	Preterm	Binary variable indicating infant was born < 37 weeks gestation.
	NICU stay	Binary variable indicating infant NICU stay following birth.
	Long birth stay	Binary variable indicating if infant was hospitalized for four or more days after birth.
***Family Context***	Teen mother at first birth	Binary variable indicating mother was < 20 years of age at the birth of her first child. Young mother at first birth has been found to be a strong predictor of poor health and social outcomes.[47]
	Lone parenthood^a^	Binary variable indicating mother was *not* married or common-law.
	Socio-economic Factor Index (SEFI2) Score^†^^b^	Composite index including four area-level measures: unemployment rate (age 15+); average household income (age 15+); proportion of single parent households; and proportion without high school graduation (age 15+). Area-level income measures have been found to be a valid proxy for individual income.[48–50] A higher SEFI2 score reflects a lower socio-economic status.^c^

^†^Measured for each time period and overall study period.

^a^ Marital status was used as a proxy measure for lone parenthood.

^b^ Created by MCHP (http://mchp-appserv.cpe.umanitoba.ca/viewDefinition.php?definitionID=103983).

^c^ For the overall study period (MDAD recurrence and severity models), values for the year before EDI period were used.

### Features of MDAD assessed

Maternal mood and anxiety disorders (MDAD) was measured by the number of physician visits for depression or anxiety (ICD-9 codes 296, 300, 309, 311) and the number of prescriptions filled for antidepressants (ATC code N06A) and sedatives/hypnotics (ATC codes N05B and N05C). Three features of MDAD were assessed in this study:

#### Timing

The relationship between child exposure to MDAD and EDI outcomes was assessed separately for each of the four time periods.

#### Recurrence

Recurrence or persistence of MDAD was measured by a non-latent variable which identified the *number of time periods* a mother had one or more contacts (prescriptions or physician visits) for MDAD for the overall study period: none (38.98%), one (27.60%), two (18.95%), three (9.88%) or all four (4.59%) time periods. The presence of MDAD in two or more periods indicated recurrence.

#### Severity

MDAD severity was measured using a non-latent variable *based on the total number of contacts* (physician visits and filled prescriptions) a mother had for the overall study period. Five categories of severity, based on the distribution of mothers’ total number of contacts, were: none (38.98%), low (14.80%), low-mid (20.80%), mid-high (15.19%) and high (10.23%).

### Analysis

This study used the two-step structural equation modeling (SEM) approach recommended by Anderson and Gerbing.[51] To assess timing, models were run on the full sample (N = 18,331) for each time period and MDAD recurrence and severity models were run for the overall study period. Confounding was controlled for through inclusion of the range of health and social variables described above, and child sex and child age.

### Conceptual model

**Fig 1:**
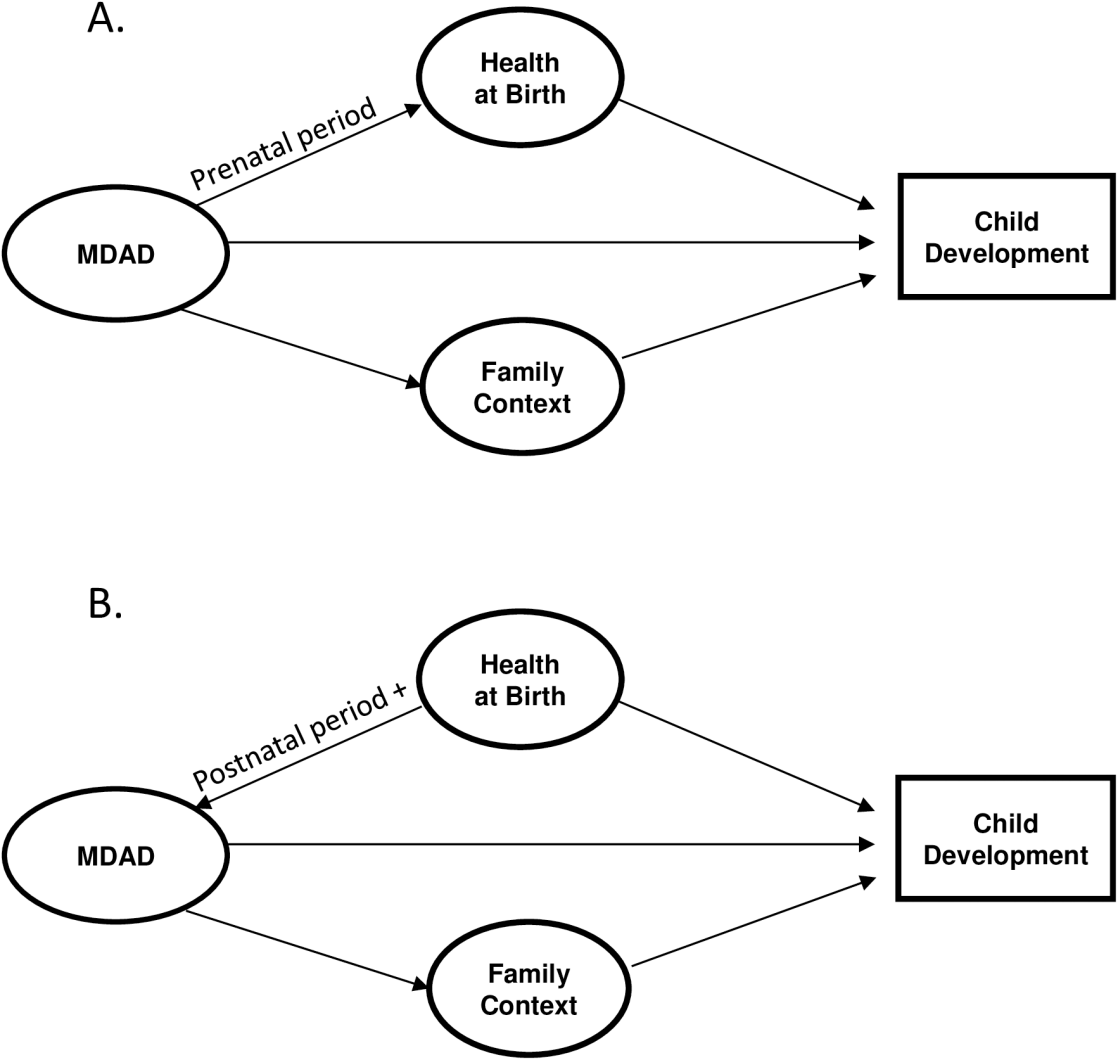
Conceptual model. (A) Model for the prenatal period with path from MDAD to Health at Birth. (B) Model for time periods following birth with path from Health at Birth to MDAD.

The theoretical model for this study was based on work by Elgar et al.[25] who developed a simplified version of the Goodman and Gotlib[52] integrative model of the transmission of risk from depressed mothers to children. In the Elgar et al. model, three key sets of mediating mechanisms are situated along the pathway between maternal depression and child adjustment problems: *biological* (e.g., genetics, in utero influences), *psychosocial* (e.g., attachment, family functioning) and *social capital* (e.g., income, social resources). In their model, the authors note that biological mechanisms mediate influences from mother to child and psychosocial and social capital mechanisms "mediate transactional influences on maternal depression and child adjustment problems." The models used for this study (Fig 1A–1B) were adapted to reflect variables available in the administrative datasets and modified based on the results of confirmatory factor analysis: In place of biological mechanisms, Health at Birth includes key birth outcomes and, in place of psychosocial and social capital mechanisms, Family Context represents key indicators of familial and economic vulnerability. The models illustrate the following direct pathways: a) Health at Birth to the outcome; b) MDAD to the outcome; and c) Family Context to the outcome. Indirect pathways include: a) MDAD to the outcome through Family Context; b) MDAD to the outcome through Health at Birth (prenatal period, Fig 1A); and c) Health at Birth to the outcome through both MDAD and Family Context for the time periods following birth (Fig 1B). For the prenatal period (Fig 1A), it was hypothesized that the path would run from MDAD to Health at Birth, to account for exposure to MDAD in utero. (Bidirectional pathways were tested in the modeling but were not a fit with the final models. We have noted this as a limitation of the study.)

Panel A illustrates the model for the prenatal year with the path running in one direction from MDAD to Health at Birth. This accounts for prenatal MDAD temporally preceding birth and the possible influence of MDAD in utero on birth outcomes. Panel B illustrates the direction of the pathway running in one direction from Health at Birth to MDAD and applies to all other models assessed. This is to account for Health at Birth temporally preceding MDAD in the time periods postnatal and beyond and the possible influence of a child's birth outcomes on MDAD.

### Research questions

The following research questions were addressed in this study:

1. Does MDAD have an impact on child development measured at school entry?
2. What aspect of MDAD–*timing*, *recurrence or severity*–is most strongly associated with child development?
3. Do Health at Birth and Family Context *mediate* the relationship between MDAD and child development?

### Model fit and statistical significance

Fit statistics used for the final models were Bentler’s Comparative Fit Index (CFI) and Bentler-Bonnet Non-Normed Fit Index (NNFI), incremental or comparative fit indices which assess model fit improvement when compared to a baseline, typically the null model[53] (values of .90 or better indicate adequate fit[54]) and the Root Mean Square Error of Approximation (RMSEA), an absolute index which compares a hypothesized model to the sample data and assesses whether the specified model reproduces patterns in the data (a threshold of < .06 was used for this study).[54] All final models in the study met or exceeded these values. In addition, due to the increased chance of a type I error with very large samples, the minimum threshold for statistical significance was p < .01.

Statistical modeling was conducted using SAS PROC CALIS in SAS version 9.3.[55] Ethics approval and privacy access were obtained from the Health Research Ethics Board (HREB) at the University of Manitoba and the Health Information Privacy Committee (HIPC) of the Government of Manitoba (HIPC# 2010-2011-33).

## Results

### Descriptive data

As illustrated in Table 4, more than one-half (52.03%) of the women in the study had at least one physician visit for depression or anxiety disorders. Nearly one-third (30.71%) filled a prescription for an antidepressant, 14% had a higher than average daily dose of antidepressants and one-quarter (24.15%) had at least one filled prescription for a sedative/hypnotic. The percentage mothers with one or more prescriptions or physician visits was lower for the prenatal and postnatal periods; the same pattern was found for the mean number of contacts per mother, particularly for number of prescriptions filled. These lower values in the prenatal and postnatal periods may be partly due to the shorter time frame (12 months) compared to the toddler period (mean = 3.7 years) and overall study period (mean = 6.6 years); however, when compared to the 12-month year before the EDI period, these lower prenatal and postnatal values may suggest lower incidence of treatment (prescriptions and physician visits) for MDAD before and after the child's birth, as was found in a large Medicaid study,[56] or higher treatment incidence in year before school entry. Two of the three family context indicators were measured across time and these show a trend toward fewer mothers lone parenting and improved SES over time.

**Table 4:** Descriptive statistics for variables measured at each time period.

Variable	Prenatal year (12 mos.)	Postnatal year (12 mos.)	Toddler years (avg. 3.7yrs)	Year Before EDI (12 mos.)	Overall study period (avg. 6.6 yrs)
	N = 18,331	N = 18,331	N = 18,331	N = 18,331	N = 18,331
	% or mean (S.D.)	% or mean (S.D.)	% or mean (S.D.)	% or mean (S.D.)	% or mean (S.D.)
**MDAD**					
***Antidepressants***					
Mothers with 1+ antidepressant Rx	4.80%	8.38%	24.45%	14.93%	30.71%
Mothers with > 1.0 ddd antidepressant Rx	1.93%	2.92%	9.01%	6.36%	14.12%
Mean number of antidepressant Rx per mother ^a^	3.37 (3.65)	3.95 (4.14)	10.25 (14.13)	6.05 (6.18)	12.71 (19.43)
***Sedatives/hypnotics***					
Mothers with 1+ sedative/hypnotic Rx	3.82%	5.05%	17.47%	9.02%	24.15%
Mean number of sedatives/hypnotic Rx per mother ^a^	2.26 (3.39)	2.95 (4.81)	5.47 (14.01)	4.07 (6.93)	6.45 (18.37)
***Physician visits***					
Mothers with 1+ depression or anxiety visits	15.47%	15.27%	39.23%	19.25%	52.03%
Mean number of depression or anxiety visits per mother ^a^	2.08 (3.22)	2.68 (4.90)	4.88 (8.52)	2.96 (4.56)	6.18 (11.59)
**Family Context Variables**					
Lone parenthood^b^	50.80%	47.20%	45.70%	45.10%	45.10%
Average SEFI2 score^c^	0.16 (0.98)	0.15 (0.99)	0.13 (1.00)	0.05 (1.00)	0.12 (0.89)

^a^ For mean number of prescriptions and physician visits, only mothers with one or more contacts are included as the large number of zero values skewed the mean values.

^b^ For the overall study period (MDAD recurrence and severity models), lone parenthood was measured at the beginning of the year before EDI.

^**c**^ Scores for the prenatal, postnatal and toddler periods were based on the 2001 Canada Census and scores for the year before EDI were for based on the 2006 Canada Census, when distribution of the dissemination areas (DAs) changed. Mean score for the overall study period were calculated by adding mean scores for each time period (measured at the beginning of the time period) and dividing by four.

Table 5 provides a summary of those study variables measured at only one time period. For EDI scores, mean values were highest for the physical health domain (8.71) and lowest for the communications skills domain (7.64). Domain scores are typically skewed toward the upper end as the vast majority of children have high scores.[57] Mean scores for this study sample are very similar to the national EDI normative sample.[57]

**Table 5:** Descriptive statistics for model variables measured at one time.

Variable	N = 18,331
	%or mean (S.D.)
**Health at Birth**	
Preterm (<37 weeks)	7.70%
Low birth weight (<2500g)	5.20%
NICU stay	4.70%
Long birth stay (4+ days)	8.90%
**Family Context**	
Mom < 20 yrs at 1st birth	8.00%
**Control variables**	
Male child	50.90%
Child age (in months)	67.91 (3.51)
**EDI scores**	
Communication Skills and General Knowledge	7.64 (2.62)
Emotional Maturity	7.91 (1.56)
Language and Cog Dev	8.12 (2.04)
Physical Health	8.71 (1.43)
Social Competence	8.27 (1.86)

### Results of structural equation models

#### Summary of models

For each EDI domain, models were run for each time period, MDAD recurrence and MDAD severity (see S1 Table, Supporting Information). Modest significant associations (-0.03, p < .01 to -0.04, p < .001) were found between prenatal MDAD and the EDI scores for all five domains (see S1 Fig, Supplementary Information). The strongest associations were found for the social, emotional and physical domains (-0.04, p < .001) and this pattern was consistent for all other time periods (-0.03 to -0.05, p < .001). Slightly lower associations between MDAD and these three outcomes were found in the postnatal year and year before the EDI (see S2–S4 Figs, Supplementary Information). For MDAD severity (see S5 Fig, Supplementary Information), a higher number of physician visits and filled prescriptions over the study period was associated with lower EDI scores and had a modest negative association with child social, emotional and physical development (-0.05, p < .001). MDAD recurrence was negatively associated with all five outcome areas (-0.02 to -0.03,p < .01 and -0.05 to -0.06, p < .001) with slightly stronger coefficients than prenatal MDAD (with the exception of communication skills). While the association between MDAD and Family Context was positive across all time periods (range = 0.04 to 0.07, p < .001, this relationship was stronger for recurrent MDAD (0.14, p < .001) and MDAD severity (0.15, p < .0.001), suggesting that the family context may be vulnerable where MDAD is present, particularly if recurrent or severe. As MDAD recurrence had a stronger relationship to the study outcomes than timing or severity, and the social competence was most negatively affected, this model is described in more detail below (see MDAD Recurrence Model).

While MDAD was negatively associated with child outcomes in all models tested, Family Context had a stronger direct relationship to all outcome domains (range = -0.21, p < .001 for emotional maturity to -0.37, p < .001 for language and cognitive development for recurrent MDAD). A modest negative association (-0.03, p < .01) was found between MDAD and language and cognitive development for prenatal and recurrent exposure; however, there was a stronger association between Family Context and child language development, particularly for the postnatal period (-0.39, p < .001). This suggests that while child language and cognitive competencies do not appear to be particularly sensitive to MDAD, there is some influence from the family environment.

Health at birth was negatively associated with EDI outcomes across all models tested (-0.05 to -0.09,p < .001), particularly for physical health (-0.09, p < .001). For the prenatal year, Health at Birth partially mediated the relationship between MDAD and child outcomes and, for all other time periods, Health at Birth was indirectly associated with the outcomes through MDAD and Family Context (see S1 Table, Supplementary Information).

The proportion of variance explained by all study variables across models tested ranged from 8.8% to 11.2% for Communication Skills to 17.6% to 20.0% for Language and Cognitive Development (see S2 Table, Supplementary Information). These R^2^ values are comparable to values found in other population-based studies that have used structural equation modeling to examine child outcomes.[18, 19]

#### MDAD recurrence model

MDAD recurrence had a stronger negative association with child outcomes than any time period or MDAD severity, suggesting that prolonged or recurrent exposure to MDAD has a stronger influence on school readiness at kindergarten than timing of exposure. Consistent with the time periods, the strongest negative associations were found for the social (-0.06, p < .001), emotional (-0.05, p < .001) and physical (-0.05, p < .001) domains, and these coefficients were slightly stronger in the MDAD recurrence model than for any other model tested in this study, suggesting that these three areas of child development are particularly vulnerable to ongoing or recurrent exposure to MDAD.

**Fig 2:**
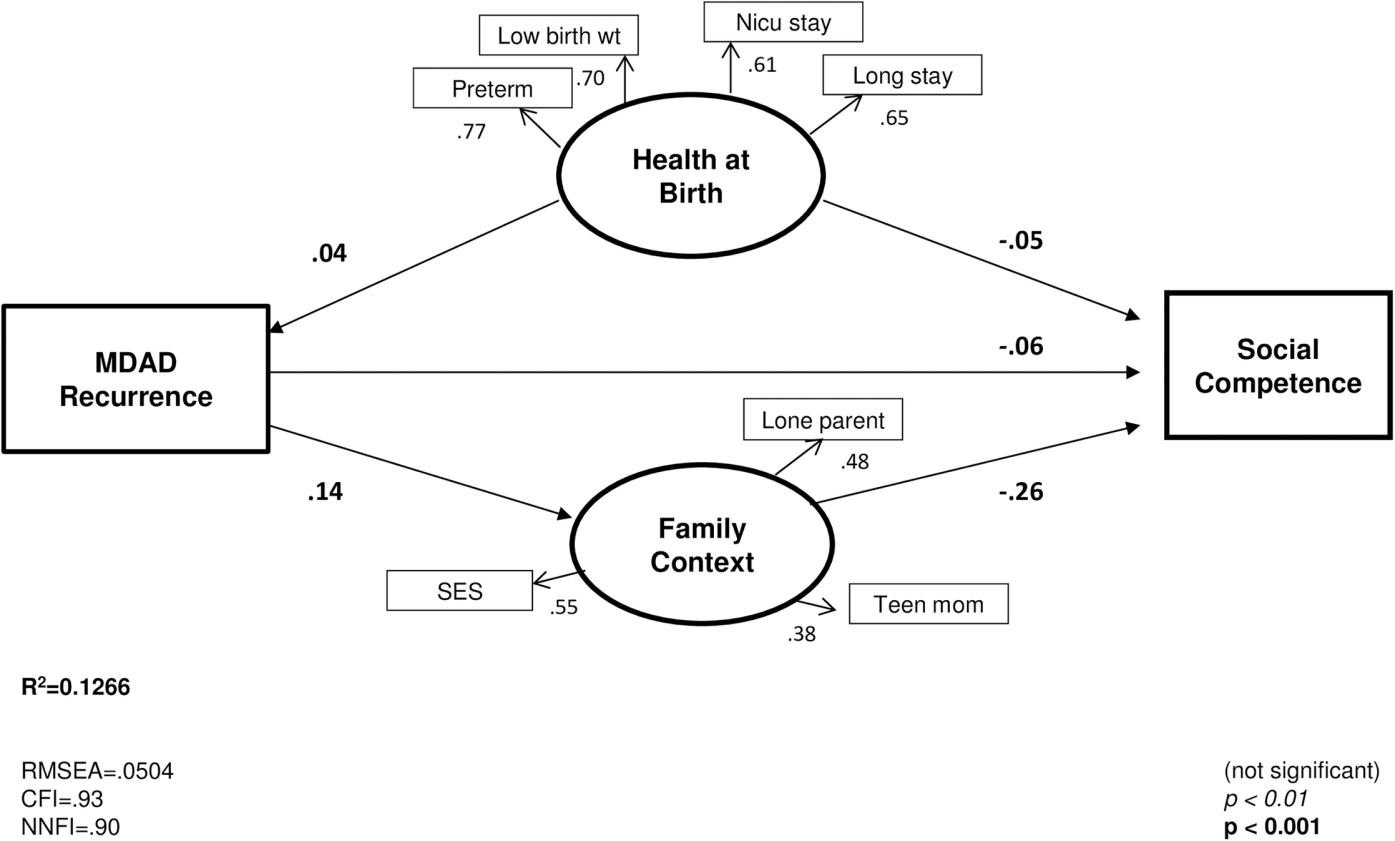
Structural equation model for recurrent MDAD and social competence at kindergarten.

Fig 2 illustrates the standardized coefficients for the relationship between recurrent MDAD and the social competence domain of the EDI. Fit statistics indicate good model fit (CFI = 0.93; NNFI = 0.90; RMSEA = 0.05). We found that the number of time periods a mother had MDAD was negatively associated with child social competence at kindergarten (-0.06, p < .001). A direct association was found between recurrent MDAD and Family Context (0.14, p < .001) and between Family Context and child social competence (-0.26, p < .001). The significant indirect association between recurrent MDAD and social competence (-0.0369, p < .001) indicates partial mediation through Family Context (see S3 Table, Supplementary Information). By dividing the direct effect of recurrent MDAD (-0.06) by the total effect (-0.0922, p < .0.001), we find that the direct effect of recurrent MDAD accounted for about two-thirds (65.1%) of the total effect, with the remainder explained by Family Context. In addition, poor Health at Birth was positively associated with MDAD recurrence (0.04, p < .001) and negatively associated with child social competence (-0.05, p < .001). The significant indirect association between Health at Birth and social competence (-0.0032, p<0.001) indicates that some of the effect of Health at Birth in the recurrent model is partially mediated through MDAD and Family Context, though only a small proportion, as most of the effect was direct (-0.0505/-0.0537 = 94.04%).

## Discussion

This study is the first to use a large sample of linked population-based data to examine the relationship between child exposure to MDAD over early childhood and five areas of child development measured at kindergarten using the Early Development Instrument. The influence of three key features of MDAD–timing, recurrence and severity–were assessed, controlling for Health at Birth and Family Context. Our findings demonstrated that MDAD was negatively associated with at least three areas of child development for all models tested; however, MDAD recurrence had a stronger negative association with outcomes than either timing or severity. In addition, this study found that challenges faced in the family context (teen mother, lone parent, low SES) had a much stronger relationship with child development scores and mediated some of the relationship between MDAD and EDI scores.

One of the unique strengths of this study was the ability to examine MDAD over four different time periods and the relationship between timing of exposure and child outcomes. A negative association between MDAD and child EDI scores was found across at least three developmental domains for the prenatal, postnatal, toddler and year before EDI periods. We found a stronger negative association with outcomes for prenatal exposure than any other time period suggesting a sensitive period for child development. This finding is consistent with other studies that found the prenatal period to be sensitive[5, 58] however, our study makes the unique contribution of a very large population-based dataset and continuous measurement of MDAD over the study period that is not subject to recall bias. It is unclear in this study what particular feature(s) of the prenatal year may account for the stronger association with EDI outcomes. It is possible that women consuming antidepressants during pregnancy had greater illness severity, thereby confounding timing with severity. Contrary to what other studies [29, 59]have found, the postnatal period did not stand out as particularly vulnerable to MDAD; this may be due to lower numbers of MDAD health care contacts for this period, though we would then expect to see stronger relationships between MDAD and outcomes at later periods with higher numbers of contacts.

In addition to timing of exposure, this study was able to examine recurrence and severity of MDAD. MDAD severity, measured by the total number of physician visits and prescriptions, was more negatively associated with EDI outcomes than exposure during any individual time period, and a dose-response relationship was found. However, exposure to recurrent MDAD over time had a stronger negative relationship, suggesting it is the number of time periods a child is exposed to MDAD, rather than total number of physician visits and prescriptions, that is most influential in our study. Other studies have found a negative relationship between chronic or recurring maternal depression or anxiety and child outcomes,[31, 58, 60, 61] however, our study was able to measure exposure continuously rather than points in time. In addition, we found that recurrent MDAD was negatively associated with all five outcome domains, particularly child social, emotional and physical development at age five. Other studies have found negative influences of maternal depression or anxiety on social and emotional outcomes[58, 59, 62] and physical development; [5, 63] however, our study is unique in that we were able to examine the effects of MDAD on five key areas of child development. It has been suggested that recurrent or persistent MDAD is more detrimental to child outcomes at school entry than other features such as timing or severity because it may be that the length of exposure, rather than timing, is most critical[23, 59] and prolonged depression or anxiety may interfere with a mother’s ability to respond to her child(ren) sensitively and consistently over time.[27] The finding of negative outcomes for children exposed to both prenatal *and* recurrent maternal depression or anxiety is consistent with other research[58] and this finding may support the programming hypothesis;[16] prenatal and long-term exposure may both contribute to an impaired child stress response and this, in turn, may lead to later illness. Modest associations were found between MDAD and child outcomes in this study and this is consistent with findings of a large meta-analysis.[13]

The associations between MDAD and EDI scores were largely mediated through other constructs; Health at Birth (for the prenatal period) and–most substantially–through Family Context (teen mother, lone parent and low SES). Other studies have found family environment or contextual risk factors to mediate the relationship between MDAD and child development.[24, 26, 60] In our study, family context had a stronger negative relationship to child language and cognitive development than other domains and this is consistent with other Manitoba research that found low SES [18] and family risk[64] to be associated with lower EDI scores for this domain. The strong influence of family/social factors has been found to persist past school entry through adolescence.[65] It may well be, as others have suggested,[24, 60] that MDAD and contextual factors work to reinforce one another; vulnerability on one negatively influences the other in an ongoing cycle.

### Strengths and limitations

There are several strengths of this study. Using population-level administrative data provided a very large sample of mother-child dyads; this resulted in a high level of power to detect whether child exposure to MDAD was negatively associated with child development at a high (p < .01 or greater) level of significance. Further, use of administrative data allowed for inclusion of vulnerable groups who may be excluded or lost to follow up in surveys (which can be as high as 40% [66]) or due to illness severity, (as high as nearly one-quarter of the sample[67]) and eliminates recall bias.[47] In addition, information from a variety of health and social administrative datasets can be linked together to examine the influence of individual, family and area-level factors on child development.[64] Other strengths of this study are the MDAD measurement and longitudinal study design. We used physician visits for depression and anxiety and filled prescriptions for antidepressants and sedatives/hypnotics to develop a unique measure of MDAD status across four discrete time periods, from the prenatal period to the EDI assessment. The longitudinal study design enabled us to measure exposure to MDAD continuously over the six-year study period rather than limited to finite points in time or large gaps between measures. This allowed for the detailed examination of timing, severity and recurrence of MDAD. Use of the population-based EDI as an outcome measure allowed for examination of the relationship between MDAD and five areas of child development. The sophisticated statistical analysis using SEM with latent constructs and the multi-level health and social measures were additional strengths in this study.

Study limitations include the challenges of using administrative data and exclusions from the study models. We used administrative data to measure MDAD, however, maternal symptoms and functioning were not available in these data. While all physician visits and filled prescriptions for depression or anxiety were identified over the six-year period, it is difficult to determine whether large numbers of visits or prescriptions are reflective of well-treated illness or greater illness severity; it is likely that the administrative data capture both of these groups. However, our finding of a dose-response relationship between MDAD severity and child outcomes suggests that higher numbers of physician visits or prescriptions may indicate more severe illness. Our study found that prenatal MDAD had a stronger negative association with child outcomes than other time periods. For the prenatal period, there were lower numbers of antidepressant prescriptions filled; it may be that those mothers who continued with antidepressant medication during pregnancy had more severe illness, thereby confounding timing with severity. Sorting out the independent influence of timing, chronicity or recurrence and severity is a challenge as these are confounded with one another.[7, 31, 61] In addition, while physician visits and filled prescriptions are indicators of “treatment prevalence,” true prevalence may be underestimated as not everyone who experiences MDAD contacts the health care system. In Manitoba, only psychotherapy provided by psychiatrists or family physicians is available in the physician billing database. Psychotherapy delivered by other providers such as psychologists, nurses, social workers, occupational therapists and other varieties of therapists is not available in the dataset. The availability of only three-digit ICD-9 codes for depression and anxiety may have overestimated prevalence of MDAD as related conditions distinguishable at the four-digit level (e.g., bipolar disorder, hypochondriasis) may be included. However, as comorbidity in mental illness is high, we expect that any contact with the health care system for one of these related conditions may confer some risk. Despite this limitation, previous Manitoba population-based research[63] found that a similar definition using physician visits for depression or anxiety and filled prescriptions for antidepressants or anxiolytics (n = 454) to be specific (83%, 95% CI, 78–87%) but not sensitive (21%, 95% CI, 16–27%) when compared to maternal report; however, sensitivity increased to 42% (95% CI, 21–66%) where mothers reported depressive symptoms more often.

Obstetric complications were not included as covariates; it is difficult to distinguish specific complications at the three-digit ICD-9 level and the Health at Birth measures (low birth weight, preterm, NICU stay and long birth hospitalization) likely capture both maternal and perinatal complications. Biological or genetic factors in mothers and children could not be accounted for and maternal behaviours such as diet and substance use (e.g., alcohol, tobacco, drugs) were not available. Such measures may account for unexplained differences in child outcomes and could assist in identifying specific mechanisms between exposure to MDAD and child outcomes. In addition, mother-child interactions were not available for this study. These interactions have been found to mediate the relationship between MDAD and child outcomes.[21, 23] Fathers were not included in this study as it is more difficult to link children to fathers in these data. However, research has found maternal depression largely accounts for the impact of parental mental illness, even after fathers' depression was controlled for.[68, 69] Previous history of depression or anxiety may be an important confounder, however, we did not include this. Research using administrative data has found the majority of women identified as having depression or anxiety at one period around pregnancy (pre-pregnancy, prenatal and postnatal) were also identified at another time period,[70] so mothers with a previous history are likely included. In the statistical models, bi-directional pathways were not examined as we were unable to obtain model fit when they were included. As others have noted, these are important to consider to determine mechanisms of how MDAD is related to child outcomes.[25, 71, 72] Despite these limitations, our study was able to identify important patterns in the relationship between MDAD and five areas of child development while overcoming limitations of small sample sizes, lost to follow-up, periodic measurement and self-report.

## Conclusions

We found that early childhood exposure to MDAD, particularly when it is recurrent, is negatively associated with five key areas of child development as measured during the child's kindergarten year. Recurrent exposure had a stronger relationship with child outcomes than MDAD severity or exposure at each of four time periods. For timing, the prenatal period had a slightly stronger association with the outcomes than other time periods. However, the study also documents that family context (teen mother, lone parent, low SES) had a much stronger association with child outcomes than MDAD. The findings from this study have implications in the clinical, program and policy arenas. Interventions and supports for mothers with depression and anxiety disorders should begin in the prenatal period and continue through early childhood as poor outcomes at school entry are predictive of later health and social outcomes.[33, 73] In addition, results from this study suggest that close attention should be paid to early childhood social, emotional and physical health as these areas appear most affected by MDAD. More research is needed to untangle the mechanisms through which MDAD negatively influences child development. The much stronger relationship found between the family context (i.e., teen parenthood, lone parenthood and low SES) and child development at kindergarten suggests that intervening in those areas will have a positive impact on school readiness. Evidence has shown that the benefits of early intervention far outweigh the initial cost investment.[37] A combination of policies to support mothers and families along with evidence-based quality early childhood interventions–and ongoing evaluation of such initiatives–can contribute to improved school readiness and later health and social outcomes.

## Supporting information

S1 Table Summary of path coefficients for models tested. (DOCX)

S2 Table Proportion of variance explained (R2) by model variables. (DOCX)

S3 Table Standardized coefficients for the MDAD recurrence model, by outcome domain. (DOCX)

S4 Table Covariance matrix for MDAD recurrence and social competence at kindergarten model. (DOCX)

S1 Fig Structural equation model for prenatal mdad and social competence at kindergarten. (TIF)

S2 Fig Structural equation model for postnatal MDAD and social competence at kindergarten. (TIF)

S3 Fig Structural equation model for toddler MDAD and social competence at kindergarten. (TIF)

S4 Fig Structural equation model for year before EDI MDAD and social competence at kindergarten. (TIF)

S5 Fig Structural equation model for MDAD severity and social competence at kindergarten. (TIF)
