# Supplementary material for: Maternal depression and anxiety disorders (MDAD) and child development: A Manitoba population-based study
Source: PLoS One. 2017 May 24;12(5):e0177065. doi: 10.1371/journal.pone.0177065 (PMC5443487; doi:10.1371/journal.pone.0177065)
Supplement: S1 Table — (DOCX) [file pone.0177065.s001.docx]

| **Model (N=18,331)** | Language and Cognitive | Social Competence | Emotional Maturity | Physical Health and Well-Being | Communication Skills |
| --- | --- | --- | --- | --- | --- |
| Prenatal MDAD | X | X | X | X | X |
| Postnatal MDAD | X | X | X | X | X |
| Toddler MDAD | X | X | X | X | X |
| Year before EDI MDAD | X | X | X | X | X |
| MDAD Recurrence | X | X | X | X | X |
| MDAD Severity | X | X | X | X | X |
