# Supplementary material for: Maternal depression and anxiety disorders (MDAD) and child development: A Manitoba population-based study
Source: PLoS One. 2017 May 24;12(5):e0177065. doi: 10.1371/journal.pone.0177065 (PMC5443487; doi:10.1371/journal.pone.0177065)
Supplement: S2 Table — (DOCX) [file pone.0177065.s002.docx]

| **Proportion of Variance Explained (R^2^) ^a^** | Language and Cognitive | Social Competence | Emotional Maturity | Physical Health and Well-Being | Communication Skills |
| --- | --- | --- | --- | --- | --- |
| Prenatal | 0.1942 | 0.1273 | 0.1122 | 0.1332 | 0.1009 |
| Postnatal | 0.1999 | 0.1346 | 0.1201 | 0.1389 | 0.1118 |
| Toddler | 0.1850 | 0.1236 | 0.1126 | 0.1238 | 0.0940 |
| Year before EDI | 0.1761 | 0.1209 | 0.1111 | 0.1262 | 0.0884 |
| Recurrence | 0.1888 | 0.1266 | 0.1148 | 0.1284 | 0.0967 |
| Severity | 0.1882 | 0.1259 | 0.1147 | 0.1282 | 0.0962 |

^a^ Includes all model variables
