# Supplementary material for: Maternal depression and anxiety disorders (MDAD) and child development: A Manitoba population-based study
Source: PLoS One. 2017 May 24;12(5):e0177065. doi: 10.1371/journal.pone.0177065 (PMC5443487; doi:10.1371/journal.pone.0177065)
Supplement: S3 Table — (DOCX) [file pone.0177065.s003.docx]

| **Variable** | Language and Cognitive | Social Competence | Emotional Maturity | Physical Health and Well-Being | Communication Skills |
| --- | --- | --- | --- | --- | --- |
| ***Predictors*** | | | | | |
| MDAD Recurrence --> Outcome | *-0.03* | **-0.06** | **-0.05** | **-0.05** | *-0.02* |
| MDAD Recurrence <-- Health at Birth | **0.04** | **0.04** | **0.04** | **0.04** | **0.04** |
| Health at Birth --> Outcome | **0.07** | **-0.05** | **-0.05** | **-0.09** | **-0.07** |
| MDAD Recurrence --> Family Context | **0.14** | **0.14** | **0.14** | **0.14** | **0.14** |
| Family Context --> Outcome | **-0.37** | **-0.26** | **-0.21** | **-0.29** | **-0.25** |
| ***Mediation*** | | | | | |
| Direct Effect of MDAD Recurrence | **-0.0276** | **-0.0553** | **0.0490** | **-0.0517** | *-0.0214* |
| Indirect Effect of MDAD Recurrence | **-0.0515** | **-0.0369** | **-0.0300** | **-0.0414** | **-0.0341** |
| Total Effect of MDAD Recurrence | **-0.0791** | **-0.0922** | **-0.0791** | **-0.0931** | **-0.0555** |
| Direct Effect of Health at Birth | **-0.0677** | **-0.0505** | **-0.0454** | **-0.0856** | **-0.0649** |
| Indirect Effect of Health at Birth | **-0.0028** | **-0.0032** | **-0.0028** | **-0.0032** | **-0.0019** |
| Total Effect of Health at Birth | **-0.0705** | **-0.0537** | **-0.0482** | **-0.0888** | **-0.0668** |
| ***Control Variables*** | | | | | |
| Male child --> Outcome | **-0.16** | **-0.20** | **-0.24** | **-0.14** | **-0.14** |
| Child age --> Outcome | **0.14** | **0.09** | **0.08** | **0.10** | **0.11** |

**<.001**; *<.01*; NS
