# Supplementary material for: Maternal depression and anxiety disorders (MDAD) and child development: A Manitoba population-based study
Source: PLoS One. 2017 May 24;12(5):e0177065. doi: 10.1371/journal.pone.0177065 (PMC5443487; doi:10.1371/journal.pone.0177065)
Supplement: S4 Table — (DOCX) [file pone.0177065.s004.docx]

| _TYPE_ | Variable | Social competence | Child age_mths | Lone parent | Low birth wt | Preterm | Teen mother at first birth | NICU stay | Male | Long birth hosp | SEFI2 score (avg) | Recurrrent MDAD |
| --- | --- | --- | --- | --- | --- | --- | --- | --- | --- | --- | --- | --- |
| MEAN |  | 8.27 | 67.91 | 0.45 | 0.05 | 0.08 | 0.08 | 0.05 | 0.51 | 0.09 | 0.12 | 1.13 |
| N |  | 18331 | 18331 | 18331 | 18331 | 18331 | 18331 | 18331 | 18331 | 18331 | 18331 | 18331 |
| COV | Social competence | 3.4438 | 0.5236 | -0.1295 | -0.0158 | -0.0179 | -0.0445 | -0.0127 | -0.1877 | -0.0244 | -0.2643 | -0.2024 |
| COV | Child age_mths | 0.5236 | 12.3074 | -0.0342 | -0.0004 | 0.0001 | 0.0143 | -0.0005 | 0.0444 | 0.0147 | 0.0235 | -0.0106 |
| COV | Lone parent | -0.1295 | -0.0342 | 0.2476 | -0.0024 | -0.0024 | 0.0340 | -0.0020 | -0.0010 | -0.0007 | 0.1058 | 0.0608 |
| COV | Low birth wt | -0.0158 | -0.0004 | -0.0024 | 0.0496 | 0.0354 | 0.0004 | 0.0177 | -0.0025 | 0.0260 | 0.0057 | 0.0000 |
| COV | Preterm | -0.0179 | 0.0001 | -0.0024 | 0.0354 | 0.0707 | -0.0001 | 0.0243 | 0.0016 | 0.0355 | 0.0059 | 0.0089 |
| COV | Teen mother at first birth | -0.0445 | 0.0143 | 0.0340 | 0.0004 | -0.0001 | 0.0737 | 0.0000 | 0.0008 | -0.0006 | 0.0455 | 0.0062 |
| COV | NICU stay | -0.0127 | -0.0005 | -0.0020 | 0.0177 | 0.0243 | 0.0000 | 0.0446 | 0.0027 | 0.0316 | -0.0019 | 0.0067 |
| COV | Male | -0.1877 | 0.0444 | -0.0010 | -0.0025 | 0.0016 | 0.0008 | 0.0027 | 0.2499 | 0.0035 | 0.0005 | -0.0027 |
| COV | Long birth hosp | -0.0244 | 0.0147 | -0.0007 | 0.0260 | 0.0355 | -0.0006 | 0.0316 | 0.0035 | 0.0808 | 0.0051 | 0.0147 |
| COV | SEFI2 score (avg) | -0.2643 | 0.0235 | 0.1058 | 0.0057 | 0.0059 | 0.0455 | -0.0019 | 0.0005 | 0.0051 | 0.7918 | 0.0656 |
| COV | Recurrent MDAD | -0.2024 | -0.0106 | 0.0608 | 0.0000 | 0.0089 | 0.0062 | 0.0067 | -0.0027 | 0.0147 | 0.0656 | 1.3693 |
