# Supplementary figures and images for: Maternal depression and anxiety disorders (MDAD) and child development: A Manitoba population-based study
Source: PLoS One. 2017 May 24;12(5):e0177065. doi: 10.1371/journal.pone.0177065 (PMC5443487; doi:10.1371/journal.pone.0177065)

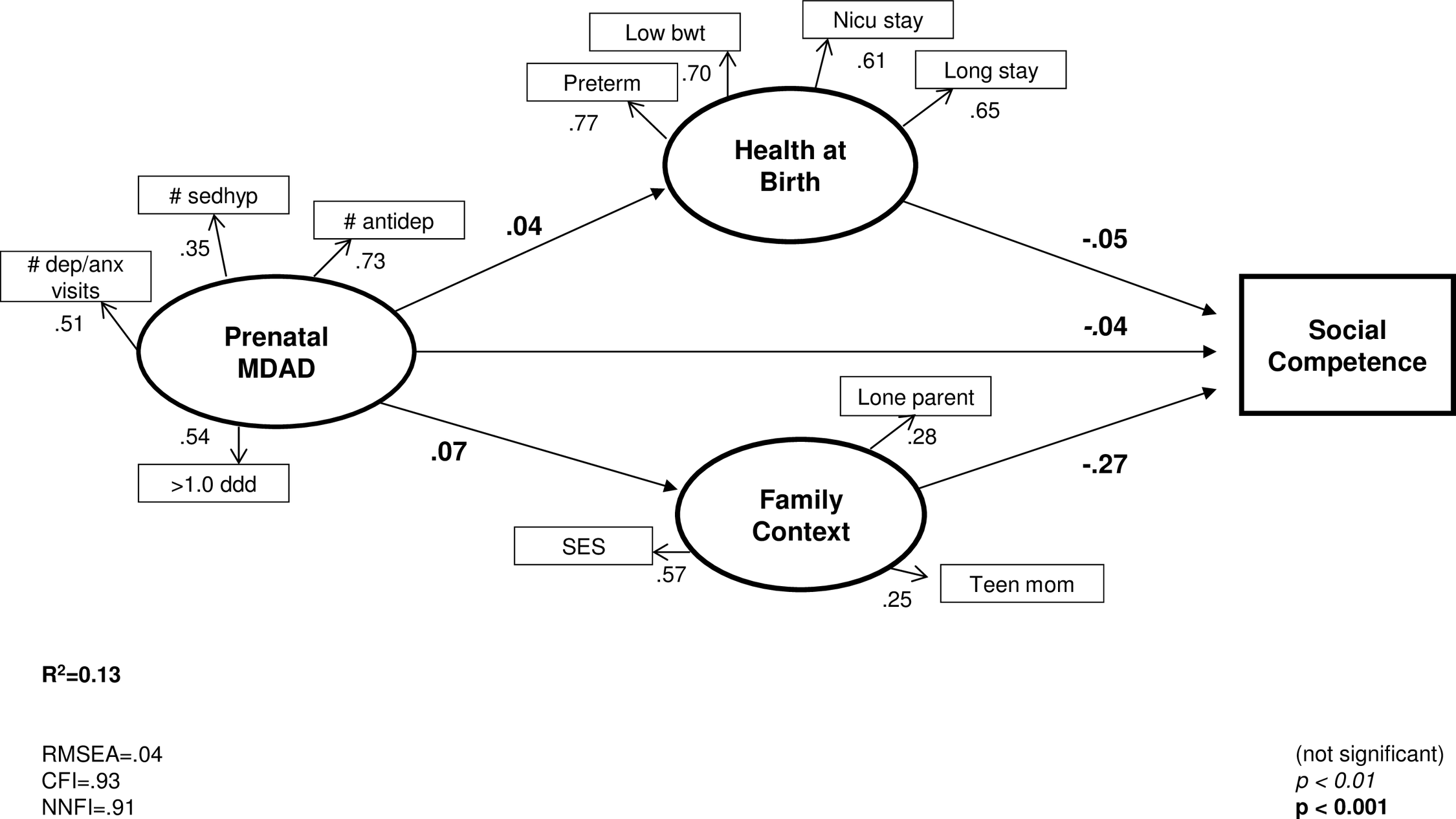

Supplement: S1 Fig — (TIF) [file pone.0177065.s005.tif]

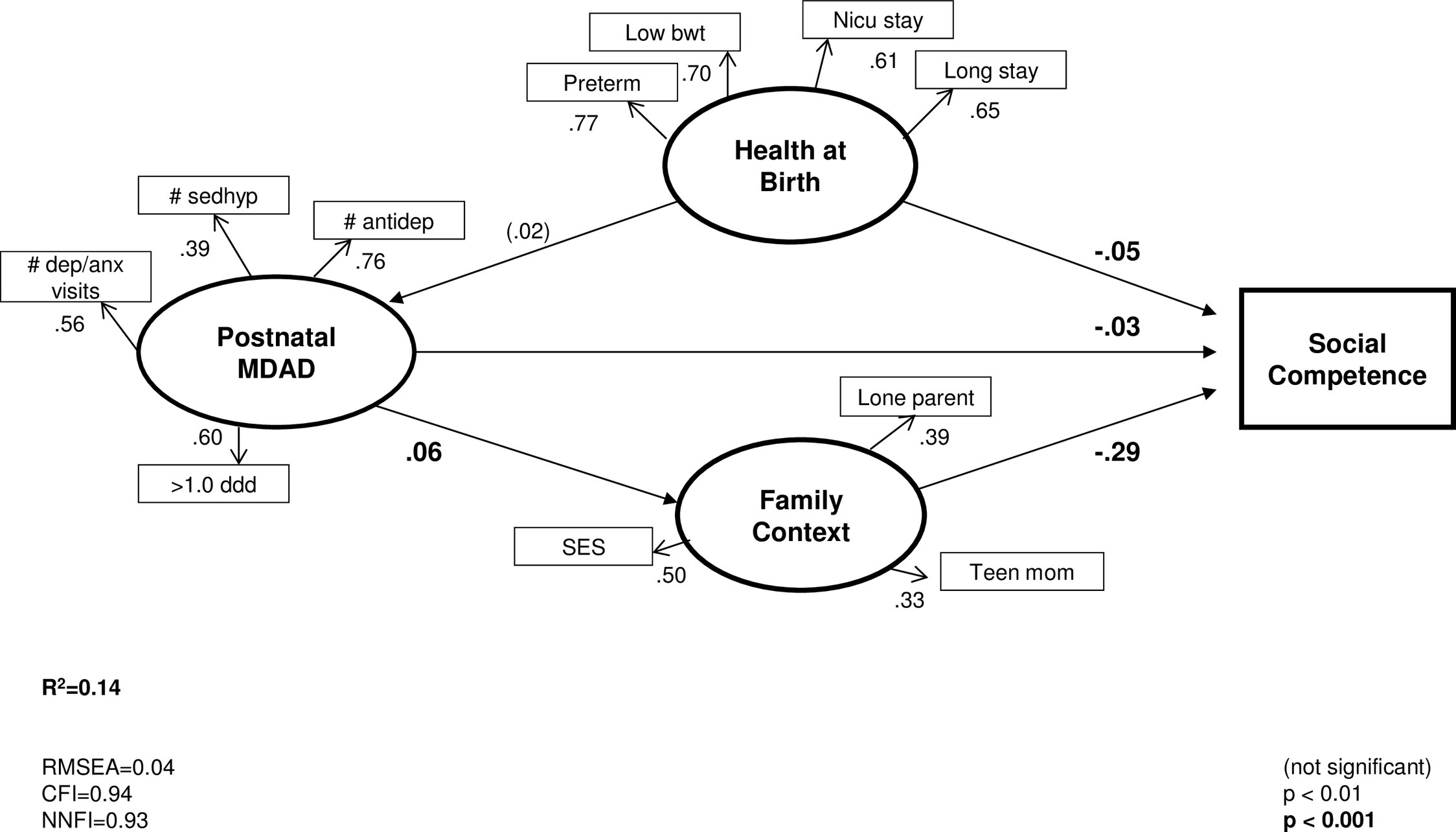

Supplement: S2 Fig — (TIF) [file pone.0177065.s006.tif]

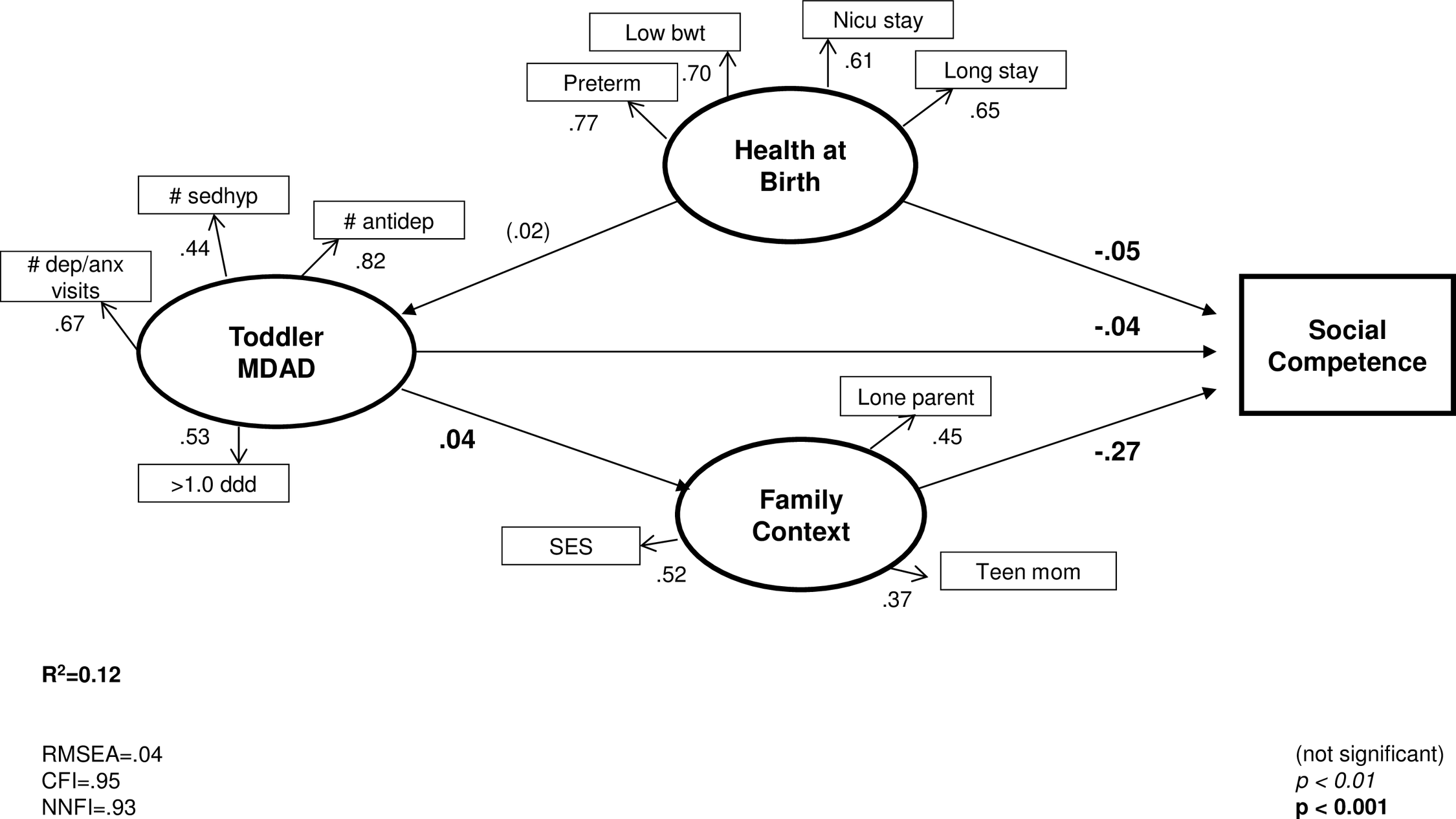

Supplement: S3 Fig — (TIF) [file pone.0177065.s007.tif]

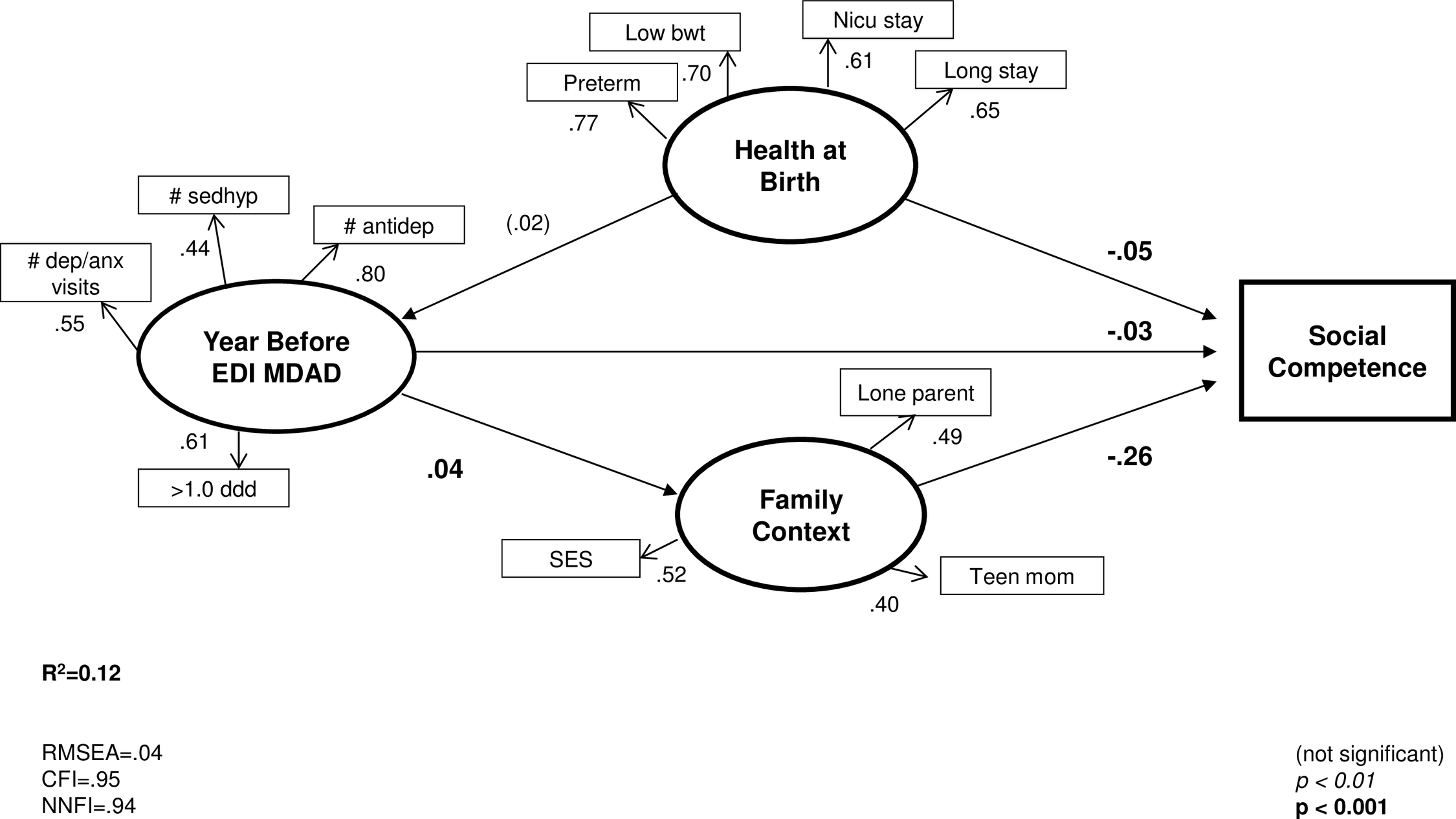

Supplement: S4 Fig — (TIF) [file pone.0177065.s008.tif]

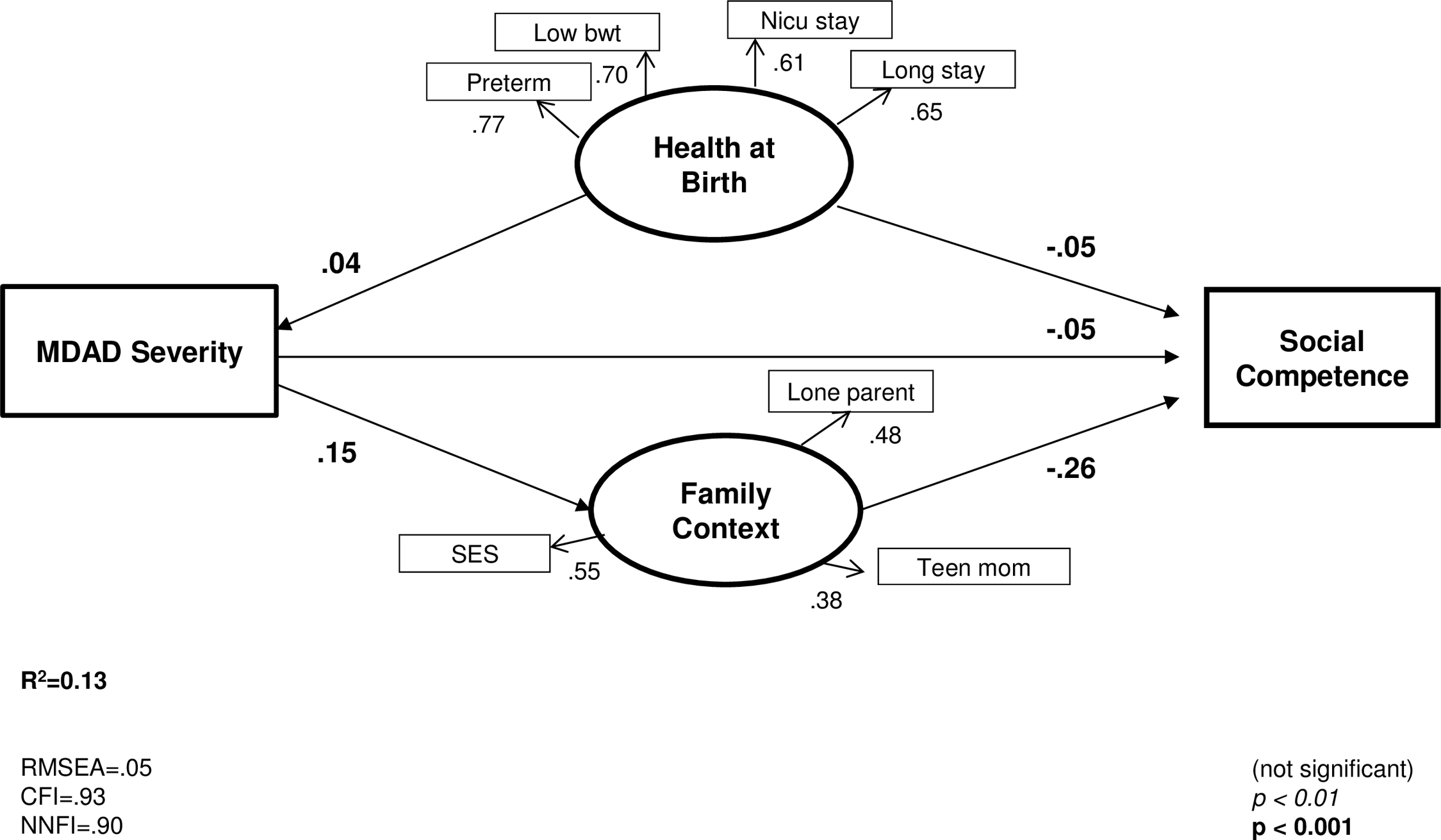

Supplement: S5 Fig — (TIF) [file pone.0177065.s009.tif]
